# Development of an Artificial Intelligence–Based Tailored Mobile Intervention for Nurse Burnout: Single-Arm Trial

**DOI:** 10.2196/54029

**Published:** 2024-06-21

**Authors:** Aram Cho, Chiyoung Cha, Gumhee Baek

**Affiliations:** 1 College of Nursing & Graduate Program in System Health Science and Engineering Ewha Womans University Seoul Republic of Korea

**Keywords:** artificial intelligence, burnout, mobile app, nurses, nurse, mHealth, mobile health, app, apps, applications, usability, satisfaction, effectiveness, tailored, mind-body, meditation, mindfulness, ACT, algorithm, algorithms, occupational health, digital health, recommender, optimization, acceptance and commitment therapy, job, worker, workers, stress, employee, employees

## Abstract

**Background:**

Nurse burnout leads to an increase in turnover, which is a serious problem in the health care system. Although there is ample evidence of nurse burnout, interventions developed in previous studies were general and did not consider specific burnout dimensions and individual characteristics.

**Objective:**

The objectives of this study were to develop and optimize the first tailored mobile intervention for nurse burnout, which recommends programs based on artificial intelligence (AI) algorithms, and to test its usability, effectiveness, and satisfaction.

**Methods:**

In this study, an AI-based mobile intervention, Nurse Healing Space, was developed to provide tailored programs for nurse burnout. The 4-week program included mindfulness meditation, laughter therapy, storytelling, reflective writing, and acceptance and commitment therapy. The AI algorithm recommended one of these programs to participants by calculating similarity through a pretest consisting of participants’ demographics, research variables, and burnout dimension scores measured with the Copenhagen Burnout Inventory. After completing a 4-week program, burnout, job stress, stress response using the Stress Response Inventory Modified Form, the usability of the app, coping strategy by the coping strategy indicator, and program satisfaction (1: very dissatisfied; 5: very satisfied) were measured. The AI recognized the recommended program as effective if the user’s burnout score reduced after the 2-week program and updated the algorithm accordingly. After a pilot test (n=10), AI optimization was performed (n=300). A paired 2-tailed *t* test, ANOVA, and the Spearman correlation were used to test the effect of the intervention and algorithm optimization.

**Results:**

Nurse Healing Space was implemented as a mobile app equipped with a system that recommended 1 program out of 4 based on similarity between users through AI. The AI algorithm worked well in matching the program recommended to participants who were most similar using valid data. Users were satisfied with the convenience and visual quality but were dissatisfied with the absence of notifications and inability to customize the program. The overall usability score of the app was 3.4 out of 5 points. Nurses’ burnout scores decreased significantly after the completion of the first 2-week program (*t*=7.012; *P*<.001) and reduced further after the second 2-week program (*t*=2.811; *P*=.01). After completing the Nurse Healing Space program, job stress (*t*=6.765; *P*<.001) and stress responses (*t*=5.864; *P*<.001) decreased significantly. During the second 2-week program, the burnout level reduced in the order of participation (*r*=–0.138; *P*=.04). User satisfaction increased for both the first (*F*=3.493; *P*=.03) and second programs (*F*=3.911; *P*=.02).

**Conclusions:**

This program effectively reduced burnout, job stress, and stress responses. Nurse managers were able to prevent nurses from resigning and maintain the quality of medical services using this AI-based program to provide tailored interventions for nurse burnout. Thus, this app could improve qualitative health care, increase employee satisfaction, reduce costs, and ultimately improve the efficiency of the health care system.

## Introduction

### Background

Burnout is a physical and mental exhaustion syndrome that includes phenomena such as a negative self-concept and work attitude and loss of interest in patients. This is a form of stress response that occurs when stress can no longer be tolerated. Burnout occurs simultaneously in 3 dimensions [1], that is, personal, work-related, and client-related dimensions [2]. The World Health Organization has defined burnout as a work-related risk factor that affects health and has included it in the 11th edition of the *International Classification of Diseases* in 2019 [3].

Nurses are known to experience high levels of burnout. The prevalence of nurse burnout has been approximately 30% worldwide in the last 10 years [4]. With the COVID-19 pandemic, nurses’ workload has increased leading to increased burnout. Nurse burnout is a serious issue in the health care system as it leads to an increase in the nurse turnover rate [5]. With increased turnover, the nursing workforce has to be replaced with new nurses, resulting in reduced work productivity and an increased burden on hospital finances [6]. In addition, burnout negatively influences the quality of nursing services and leads to frequent absences [7,8].

Acknowledging the significance of nurse burnout, various programs have been developed and tested. In a study that reviewed burnout reduction interventions for nurses and doctors, interventions such as yoga, meditation, and mindfulness reportedly alleviated burnout [9]. However, the program was conducted in person, and interventions for medical personnel can be burdensome due to time and location constraints [10]. Moreover, as nurses work in shifts, there are time and space restrictions for interventions. However, web-based interventions do not require a facilitator and are, therefore, better than in-person interventions in terms of efficiency [11].

In previous studies, interventions through mobile apps to confirm the effect of burnout were based on voice programs [12,13]. Previous studies on burnout interventions introduced one-size-fits-all programs. However, nurses experience varying dimensions of burnout. Artificial intelligence (AI)–based recommendation systems based on similarity are widely used in various fields. Netflix and YouTube recommend similar media based on individual content preferences [14,15]. In addition, in the marketing field, similar products are recommended by analyzing the commonalities of products that consumers have purchased [16]. As the accuracy and diversity of recommendations increase, user satisfaction increases [17]. Recommendation systems commonly use a user-based collaborative filtering system, which recommends items or content based on similarities between users’ interest profiles [18]. Digital Me, an AI-based product service system, measures a user’s state through user behavior analysis, predicts it, and makes recommendations. The recommendation system maximizes a specific score or finds similar but superior existing users, thus attaining its goal [19]. Therefore, a similar system could be used to provide a tailored program for nurse burnout.

Nurses’ burnout is influenced by various factors, such as their demographic and job-related characteristics [20-22]. Burnout patterns differ depending on coping strategies, whether working night shifts, working at a tertiary hospital, age, and department [23-25]. Therefore, when recommending burnout reduction programs for nurses, we could consider that similar burnout experiences are shared among individuals with similar general and job-related characteristics, rather than simply relying on user preferences or uniform delivery. The system we have developed measures the similarity between users based on demographics, research variables (job stress, stress response, and coping strategy), and burnout dimension scores.

### Literature Review

Cognitive behavioral therapy (CBT)–based interventions are evidence-based interventions to reduce burnout and stress in health care professionals and improve mental health, healthy lifestyle beliefs, and job satisfaction [26]. Several programs have been suggested to reduce and prevent nurse burnout. One of these, mindfulness meditation, is a well-known technique for managing stress and burnout. Mindfulness is the attitude of paying attention to present moment events and accepting feelings, ideas, and sensations without passing judgment [27]. Mindfulness-based cognitive therapy is a group psychotherapy program that combines mindfulness and CBT and has been proven to effectively improve depression and prevent its recurrence [28]. Mindfulness-based training is effective in reducing burnout not only in nurses [29] but also in other health care professionals [30,31]. In addition, mindfulness meditation has been reported to enhance positive cognitive retraining by increasing positive mood and brain activity [32]. A recent study demonstrated that a website and a smartphone app for mindfulness were effective in reducing burnout among health care professionals. [33].

Another therapeutic intervention is laughter therapy. Laughter therapy improves healing and coping abilities in physical, psychological, and cognitive aspects by inducing laughter, smiles, and pleasant emotions through physical and intellectual interactions with the body and the mind [34]. It is well known that laughter therapy can reduce psychosocial conditions, such as stress, anxiety, and depression, in people with health issues, such as patients with cancer, middle-aged women, and mothers [35-37]; it has also recently been reported as an effective way to improve life satisfaction and reduce burnout in nurses [38]. Using humor to deal with stressful and burnout situations can increase happiness and life satisfaction in individuals by fostering more positive evaluations [39].

Storytelling, one of the most fundamental and traditional forms of communication, can influence behavior by allowing people to express human identity and self-identify, share similar life experiences, and explain situations [40]. A form of CBT called “narrative CBT” uses storytelling [41]. The use of storytelling among nurses provides a space for nurses to talk about, listen to, perceive, and evaluate their experiences [42]. In previous studies, nurses who took part in storytelling workshops gave storytelling a positive evaluation [43]. In addition, expressive writing has been shown to improve mood, cognitive function, and mental health problems [44,45]. In a recent study that used storytelling and reflective writing as an intervention for oncology nurses, self-awareness and self-compassion reportedly increased, and insomnia and loneliness decreased [46]. Therefore, storytelling and reflective writing can improve personal well-being by providing skills to recognize one’s emotions and cope with work-related emotions.

Acceptance and commitment therapy (ACT) is part of the third wave of CBT [47]. It is a comprehensive psychological therapeutic strategy that can be used to treat a variety of problems and disorders. It incorporates acceptance and mindfulness processes, active engagement, and behavioral change processes [48]. Recently, it was provided to a group of employees in the workplace [49] and was verified as an intervention for stress and burnout management for various medical staff, including nurses [50]. It has been suggested that ACT may have the potential to reduce burnout among various occupational groups [51].

### Objective

The purpose of this study was to develop and optimize an AI-based tailored mobile intervention, Nurse Healing Space, to reduce nurse burnout. Specifically, we designed an AI algorithm that provides tailored burnout reduction programs using user similarity scores and assessed the usability of the intervention. After optimizing the AI algorithm, we tested the effectiveness of the program for burnout, job stress, stress response, coping strategies, and user satisfaction.

## Methods

### Research Overview

We developed the mobile app, Nurse Healing Space, from December 2021 to July 2022 and then conducted a pilot test in August 2022 to evaluate the usability and test the operation of the app. From September 2022 to February 2023, 300 people were recruited to accumulate data and optimize algorithms. Before starting the program, we instructed participants on how to participate. This was necessary to prevent dropout as the program’s duration was 4 weeks (pretest, program 1 first and second weeks, posttest 1, program 2 first and second weeks, and posttest 2), with a total of 7 stages. A total of 325 potential participants showed interest in the study, of whom, 300 completed the intervention.

### Nurse Healing Space

### Overview

**Figure 1:**
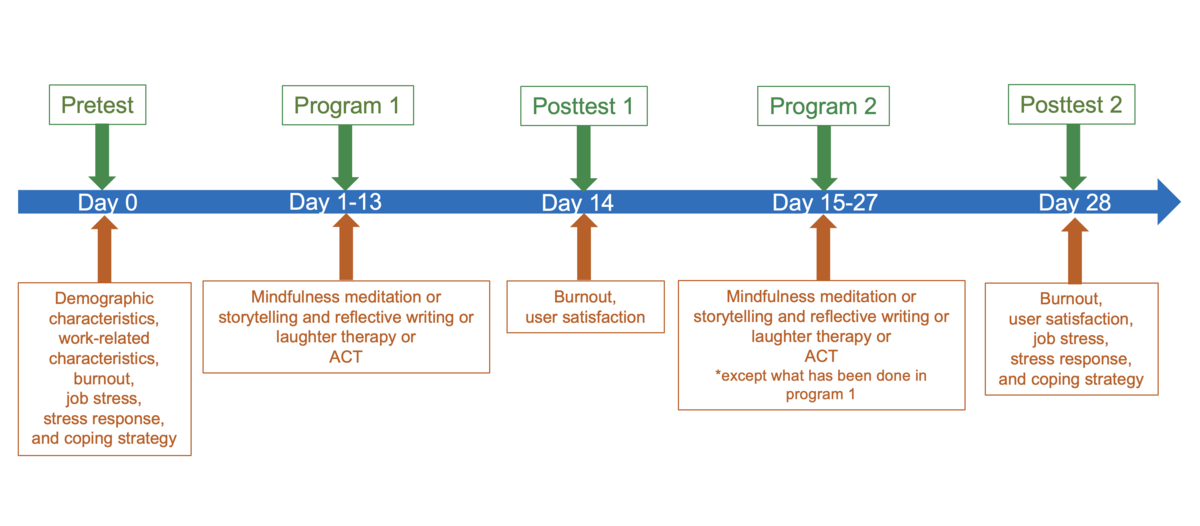
Nurse Healing Space operation process. ACT: acceptance and commitment therapy.

Nurse Healing Space is a 4-week intervention that consists of two 2-week programs to reduce burnout (Figure 1). Users fill out questionnaires (pretest), and the AI recommends 1 of 4 programs (program 1) based on the similarity of users. After the completion of program 1, users are tested for burnout and program satisfaction (posttest 1). The AI recommends a second program out of the 3 remaining programs (program 2) based on the similarity of users from posttest 1. After completion of program 2, users fill out another questionnaire (posttest 2).

**Figure 2:**
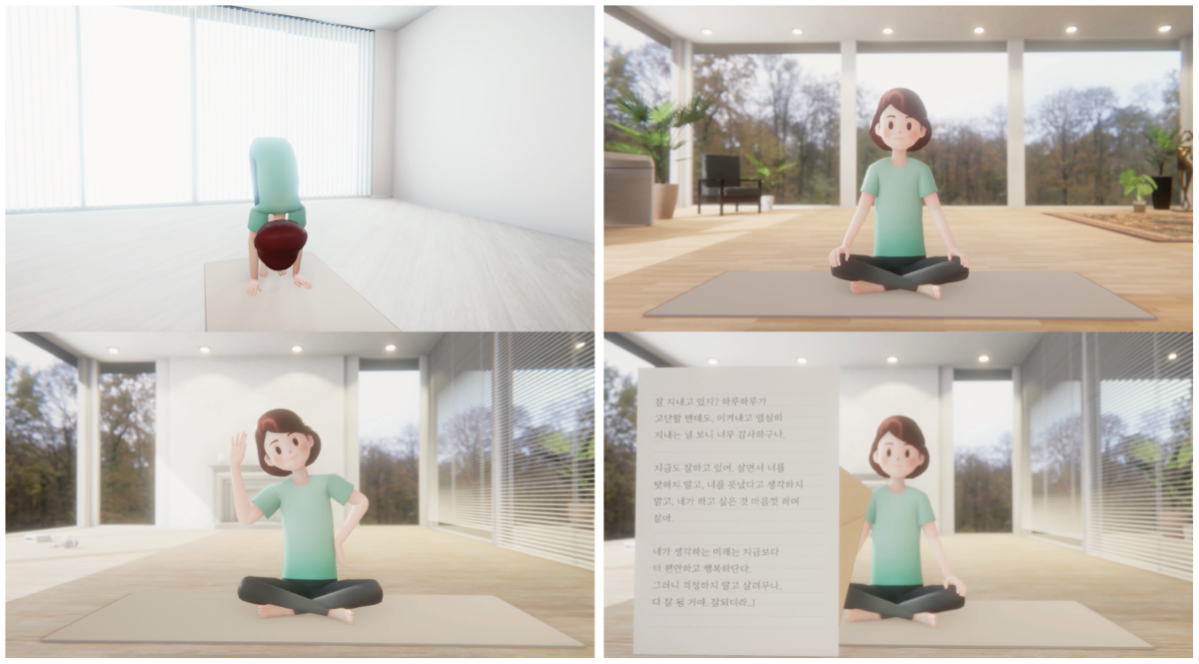
Scenes from the program.

Each program consists of 6 sessions of 10-15 minutes each. The animated female character in the video guides participants (Figure 2). All the programs were developed by the research team in consultation with experts in each field. Below are the descriptions of the four 2-week programs.

#### Mindfulness Meditation Program

Mindfulness meditation is conducted by the fictional nurse character guiding the program through narration and demonstration of movements. The contents for mindfulness meditation were organized based on Kabat-Zinn’s [27] mindfulness meditation by a researcher (CC) who had previously developed mindfulness programs and reviewed them by an expert. Each session consists of an intro, mindfulness meditation, and wrap-up. The intro explains the mindset before starting the mindfulness meditation. Mindfulness meditation is composed of a mixture of the following 2 in each session: body scan, sitting up straight meditation, relaxation meditation, yoga meditation, and self-compassion meditation. The wrap-up explains ways to apply mindfulness meditation to everyday life.

#### Storytelling and Reflective Writing Program

In storytelling, a fictional nurse narrates her story of burnout and coping strategies during each session. The topics in the sessions consist of “Work is piling up,” “I am a person too,” “There aren’t enough hours in the day,” and “Am I an emotional outlet?” The work environment is poor, with not enough nurses to take care of patients. After the storytelling, the fictional nurse asks participants to reflect on their experiences and write about them. Participants can freely share their reflective writings on the bulletin board of the app.

#### Laughter Therapy Program

For laughter therapy, a fictional nurse guides the laughter exercise with background music. Each session consists of an intro, laughter exercise, and wrap-up. The contents were organized based on the laughter therapy program of the Korean Society of Laughter Clinic and were reviewed by an expert. The introductory part consists of a brief explanation of laughter therapy and prelaughter exercises. Each laughter exercise consists of 2 exercises with exciting music and the laughter follows the rhythm. The wrap-up consists of self-expression and laughter meditation.

#### ACT Program

In ACT, a fictional nurse guides the ACT by narration and showing images for cognitive change by using various techniques, such as the breathing technique and music meditation. The contents were organized based on the ACT founder’s book [52] and reviewed by an expert. The ACT consists of “Hello my heart” (introduction), “Thinking is just thinking” (cognitive diffusion), “This is the moment” (being present in the moment), “Look back at me” (self as context), “Worthy life” (the best 3 moments of my life), and “Let’s do it together” (committed action and willingness).

### AI-Based Program Recommendation System

**Figure 3:**
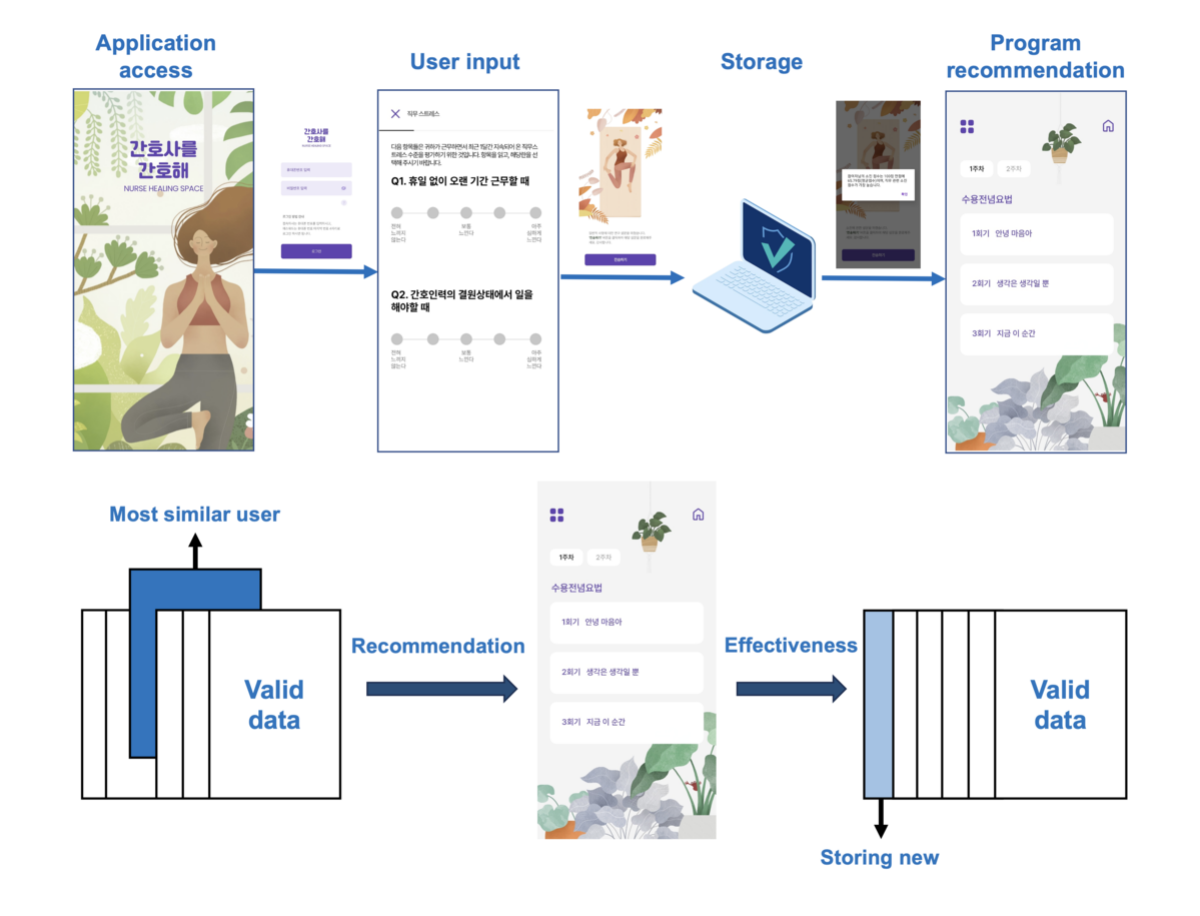
Nurse Healing Space operation.

When users fill out the pretest, their responses are calculated and stored as scores. One of 4 programs that were effective for a previous user who is most similar to the new user, is recommended to the new user. We define similar users as having similar demographic and work-related characteristics and burnout dimensions. After the completion of program 1, users are tested for burnout dimensions and program satisfaction. Thereafter, users are matched with program 2, which is 1 of 3 remaining programs that were evaluated as effective for burnout by the most similar users based on similarity scores for demographic and work-related characteristics and burnout dimensions. After the completion of program 2, users fill out posttest 2. The operation of the Nurse Healing Space app is shown in Figure 3.

#### Measuring Similarity Between Users

We used a pilot test with 10 nurses to collect initial data using the preallocated program according to the highest subdimension recommended. With 10 sets of existing data stored through the pilot test, when new users are entered, the algorithm compares demographic and work-related characteristics, job stress, stress response, and coping strategies with the stored data to find the most similar existing users with a matching subdimension of burnout. The search method is as follows.

In the recommendation system, the existing user is denoted as *i* and the new user as *j*. Zero is assigned to the element if the value of the *k* variable of the user *i* and the *k* variable of the user *j* matches, and 1 is assigned to the inconsistent element. Specifically, the similarity of each element of the categorical variable can be determined.



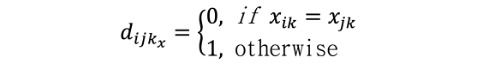

**(1)**


*d_ijk_x__* refers to the similarity of each element of the categorical variable; *i* is an existing user, *j* is a new user, and *x* is an element included in the categorical information. Thereafter, the recommendation system calculates the similarity of each categorical variable by adding the similarity of each element.

The recommendation system calculates the similarity between the users and the existing data by applying the weight of each element to the numerical variable. The numerical type is calculated by dividing the absolute value of the difference between the value of the *k* variable of user *i* and the value of the *k* variable of user *j* by the range of the *k* variable. Specifically, the similarity for each element in the numerical type of information is determined.



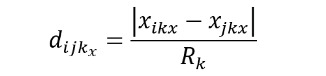

**(2)**


indicates the similarity of each element in a numerical variable, *i* is an existing user, *j* is a new user, *x* is an element included in the numerical information, and *R* may indicate the range of the variable. Thereafter, the recommendation system calculates the similarity of the numerical variable by adding the similarity for each element.

Table 1 shows that when a new user is entered, a similarity between the new user and the existing users A, B, and C is calculated. The recommendation system determines the similarity between the new user and the existing users by calculating the similarity scores from 5 categorical variables and 17 numerical variables. Similarity is based on the sum of each value. The smaller the sum of the similarity level, the higher the similarity with the new user. The possible ranges of similarity scores are between 0 and 22. For example, in Table 1, user B has the lowest similarity scores and is determined as the user that is most similar to the new user.

**Table 1:** Measuring similarity between users.

Variable			Raw score						New user		Similarity calculation				
			User A		User B		User C				User A		User B		User C
**Categorical data**															
	Sex	1		1		1		1		1		1		1	
	Marital status	1		2		1		3		1		1		1	
**Continuous data**															
	Age	30		45		25		45		0.39		0.00		0.53	
	Hospital size	5		3		4		5		0.00		0.50		0.25	
	Clinical experiences	1		1		2		3		0.40		0.40		0.20	
Similarity			—^a^		—		—		—		8.03		5.96		7.64

^a^Not applicable.

### Measurement

Burnout was measured using Copenhagen Burnout Inventory (CBI) [2], which was translated into the Korean version. Cronbach α at the time of translation was 0.93 [53]. The CBI consists of 19 items that measure 3 subdomains: personal burnout (6 items, ranging between 0 and 31.5), work-related burnout (7 items, ranging between 0 and 37), and client-related burnout (6 items, ranging between 0 and 31.5). Possible scores range between 0 and 100, with higher scores indicating higher burnout. In this study, Cronbach α was 0.94.

Job stress was measured with an instrument developed for Korean nurses [54]. This instrument consists of 23 items. The scores range from 23 to 115, with a higher score indicating greater job stress. In this study, Cronbach α was 0.92. Stress response was measured using the Stress Response Inventory Modified Form [55], which consists of 22 items. Possible scores ranged between 0 and 88, with higher scores indicating higher levels of stress responses. In this study, Cronbach α was 0.95.

Coping strategy was measured using the Coping strategy indicator [56], which was translated into the Korean version. Cronbach α at the time of translation was 0.84 [57]. The instrument consists of 33 items. The possible range of scores was 33-99. In this study, Cronbach α was 0.89.

A rating scale was used to evaluate the usability of apps [58]. This measure consisted of 23 items in 5 areas—engagement, functionality, aesthetics, information, and subjective mobile app quality. The possible range of scores was 26-130, with higher scores indicating higher usability and quality. App satisfaction was measured with 1 item using a 5-point Likert-type rating scale (1: very dissatisfied; 5: very satisfied).

#### Valid Data

Data are evaluated as valid when burnout dimension scores after program completion decrease by at least 5 points on a 0-100 scale based on the criterion suggested in a previous study [2]. Having valid data implies that the program effectively lowers burnout and data are stored in the AI algorithm.

Table 2 illustrates an example of the process of determining valid data and the program provided to each user with the change in burnout scores after program completion. For example, user A achieved a 5-point reduction in personal burnout after completion of program 1; this was classified as valid data and included in the algorithm. However, after the completion of program 2, the burnout scores of user A reduced for all burnout dimensions by less than 5 points; this was considered invalid and not stored in the algorithm. None of user B’s data were stored because user B did not have valid data. For user C, burnout scores for personal and work-related burnout decreased by 5 points after completion of program 1. For 2 burnout subdimensions the score reduced by more than 5 points. In this case, it was considered that burnout had decreased as a result of other causes, and the data were regarded as invalid. For user D, the personal burnout score reduced by 5 points after completion of program 1 and work-related burnout score by 6 points after completion of program 2; thus, their data were stored as valid data.

**Table 2:** Valid data selection criteria.

			Personal burnout score		Work-related burnout score		Client-related burnout score		Program number		Data type
**User A**											
	Pretest	45		40		39		—^a^		—	
	Reduction after program 1	5		3		4		Program 1		Valid	
	Posttest 1	40		37		35		—		—	
	Reduction after program 2	4		2		2		Program 2		Invalid	
	Posttest 2	36		35		33		—		—	
**User B**											
	Pretest	45		40		39		—		—	
	Reduction after program 1	3		3		4		Program 1		Invalid	
	Posttest 1	42		37		35		—		—	
	Reduction after program 2	2		0		2		Program 2		Invalid	
	Posttest 2	40		37		33		—		—	
**User C**											
	Pretest	45		40		39		—		—	
	Reduction after program 1	5		5		4		Program 1		Invalid	
	Posttest 1	40		35		35		—		—	
	Reduction after program 2	0		2		2		Program 2		Invalid	
	Posttest 2	40		33		33		—		—	
**User D**											
	Pretest	45		40		39		—		—	
	Reduction after program 1	5		3		4		Program 1		Valid	
	Posttest 1	40		37		35		—		—	
	Reduction after program 2	4		6		2		Program 2		Valid	
	Posttest 2	36		31		33		—		—	

^a^Not applicable.

#### AI Algorithm

As valid data are continuously accumulated, the recommendation system learns and updates the artificial neural network. The recommendation system determines the most similar user from the user information using the learned artificial neural network. The artificial neural network determines similar users using all the similarity scores of the valid data, and programs that have been effective in reducing burnout for similar users are provided to new users. Thereafter, the artificial neural network compares the degree of change in the pre- and posttest burnout scores of the new user to determine whether the program provided was effective for the new user and stores data about the user if it is determined to be valid. The recommendation system updates the artificial neural network by continuously updating valid data. Thus, the artificial neural network can acquire valid data for more users over time, and by determining users that are similar to new users, it provides more effective programs to new users over time. To ensure that the algorithm operates as intended, we extracted valid data from 10 pilot tests and 299 optimizations. Subsequently, we manually calculated the similarity score of the 300th participant using Excel and checked the suggested program and compared it with what was suggested to the user.

### Data Collection and Setting

We used 2 large web-based nursing communities with 94,000, and 170,000 members for data collection. We posted an advertisement on 2 large web-based nursing communities in August 2022 to recruit participants for a pilot test. For the pilot test, we adopted a minimum of 12 participants recommended by Whitehead et al [59]; however, only 10 participants were able to complete the pilot test. After the pilot test, we posted advertisements on 2 large web-based nursing communities between September 2022 and February 2023 to recruit participants for AI algorithm optimization. The number of participants required for measuring the intervention effect was calculated based on the paired 2-tailed *t* test for measuring the effect of burnout, a research variable of this study. Using G-power (version 3.1.9.4) with an effect size of 0.2, significance level of .05, and power of 0.95, a total of 272 people were calculated. Due to the insufficiency of previous studies on developing mobile interventions for nursing burnout, the effect size was conservatively considered at a low threshold to achieve treatment effects. A total of 325 potential participants showed interest in the study, of whom, 300 completed the study. Their data were used for AI algorithm optimization. The inclusion criteria for the participants were nurses who had been providing care independently at hospitals for the past month at the time of data collection.

### Ethical Considerations

This study was reviewed and approved by the institutional review board (ewha-202208-0005-01) at the principal investigator’s institution. Informed consent was obtained from all participants. Participants received information about the study’s goals, anonymity, confidentiality, and their right to withdraw from the study at any time before signing the online informed consent form. As compensation for their participation, participants were provided with mobile coupons valued at US $3.60. The collected data were encrypted and stored on a computer accessible only to the researchers.

### Statistical Analysis

We used software SPSS (version 23.0; IBM Corp) to facilitate data analysis. Descriptive statistics were used to report sociodemographic and work-related characteristics and study variables. All variables met the assumption for normal distribution: skewness of 1 or less and kurtosis of 5 or less [60]. A paired *t* test was conducted on burnout, job stress, stress response, and coping strategies before and after the program. Spearman correlation was performed to examine changes in research variables and burnout reduction based on data accumulation. Nonparametric analysis (Spearman correlation) was used because data accumulation is considered an ordinal variable in that it follows the order of participation. ANOVA was conducted on satisfaction change by grouping participants into 3 groups: the first 100 participants, the second 100 participants, and the third 100 participants; the Scheffe test was performed for post hoc analysis.

## Results

### Algorithm Operation

The 300th participant had the highest work-related burnout and client-related burnout among the burnout dimensions, and laughter therapy was recommended. The similarity scores between the 300th participant and all the accumulated valid data ranged between 1.17 and 8.14. It was confirmed that the program completed by the participant with the lowest similarity was laughter therapy, which was recommended for the 300th participant. The highest burnout subtype for the most similar participant was work-related burnout, and the participant’s data were classified as valid data because of the effect of laughter therapy. The calculated similarity with the participant was 1.17. To make it more indisputable, we checked whether the similarity system worked in the same way for the 299th participant, and it was confirmed that the program recommended to the most similar participant among the valid data was the same.

### Valid Data

Of the total data, 45.7% (274/600) were selected as valid data. Personal burnout was the highest subdimension with 57.4% of valid data and work-related burnout was the next highest subdimension with 25.9% of valid data, followed by client burnout with 40% of valid data including duplicates. In terms of programs, laughter therapy accounted for the largest proportion of valid data, with 32.8%, followed by 28.1% for mindfulness meditation, 20.4% for ACT, and 18.6% for storytelling and reflective writing.

### Usability Assessment

Users scored ease of use, good visual design, and high quality of content graphics more than 4 out of 5. However, users felt most uncomfortable with the absence of reminders and notification functions and the inability to interact with the app. Moreover, they were not satisfied with the fact that the app could not be set up to suit their preferences. The overall score for this app was 3.4 out of 5 on a Likert scale. Furthermore, the total mean score for each question was 3.4 out of 5 on a Likert scale.

### Intervention Effects

The mean age of the participants was 32.8 (SD 5.75) years. Of these, 97.7% (293/300) were female, 87% (261/300) were staff nurses, and 62.3% (187/300) worked in hospitals with more than 500 beds (Table 3).

The decrease in burnout was statistically significant, from 62.36 (SD 18.0) at pretest to 55.22 (SD 18.79) at posttest 1 (*t*=7.012; *P*<.001) and 52.70 (SD 20.15) at posttest 2 (*t*=8.692; *P*<.001). Reduction in burnout scores from posttest 1 to posttest 2 was also statistically significant (*t*=2.811; *P*=.01). Moreover, burnout scores decreased significantly before the program and after the first and second programs (*t*=8.692; *P*<.001). Personal burnout decreased statistically from 20.40 (SD 6.39) at pretest to 18.15 (SD 6.38) at posttest 1 (*t*=6.134; *P*<.001) and 17.30 (SD 7.33) at posttest 2 (*t*=7.535; *P*<.001). In addition, the reduction in personal burnout from posttest 1 to posttest 2 was statistically significant (*t*=2.597; *P*=.01). Work-related burnout decreased statistically from 22.22 (SD 6.39) at pretest to 19.83 (SD 6.49) at posttest 1 (*t*=6.816; *P*<.001) and 19.06 (SD 6.68) at posttest 2 (*t*=8.286; *P*<.001). The reduction in work-related burnout from posttest 1 to posttest 2 was also statistically significant (*t*=2.306; *P*=.02). Client-related burnout decreased statistically from 19.72 (SD 6.63) at pretest to 17.22 (SD 6.91) at posttest 1 (*t*=6.439; *P*<.001) and 16.32 (SD 7.15) at posttest 2 (*t*=8.329; *P*<.001). Reduction in client-related burnout from posttest 1 to posttest 2 was also statistically significant (*t*=2.704; *P*=.01).

Job stress, stress response, and coping strategy were measured twice before the program and after the first and second programs. After the completion of the two 2-week programs, the participants reported a statistically significant reduction in job stress (*t*=6.765; *P*<.001) and stress response (*t*=5.864; *P*<.001) (Table 4).

**Table 3:** General characteristics of participants (N=300).

Characteristic			Values
Age (years), mean (SD)			32.81 (5.754)
Gender (female), n (%)			293 (97.7)
**Marital status, n (%)**			
	Married	178 (59.3)	
	Single	118 (39.3)	
	Divorced or separated	4 (1.3)	
**Position, n (%)**			
	Staff nurse	261 (87)	
	Charge nurse	35 (11.7)	
	Head nurse or team leader	4 (1.3)	
**Hospital size (beds), n (%)**			
	<30	7 (2.3)	
	31-100	25 (8.3)	
	101-300	63 (21)	
	301-500	18 (6)	
	≥500	187 (62.3)	
Clinical experience (months), mean (SD)			95.83 (69.66)
**Working department, n (%)**			
	General ward	172 (57.3)	
	ICU^a^	16 (5.3)	
	Special units^b^	30 (10)	
	Outpatient or clinic or administration	63 (21)	
	Others	19 (6.3)	
**Overtime during the past month (hours), n (%)**			
	None	41 (13.7)	
	≤0.5	78 (26)	
	0.5-1	77 (25.7)	
	1-1.5	50 (16.7)	
	1.5-2	17 (5.7)	
	>2	37 (12.3)	
**Type of shift, n (%)**			
	8-hour shift	180 (60)	
	12-hour shift	18 (6)	
	Fixed	101 (33.7)	
	Others	1 (0.3)	
Turnover intention (range of score: 0-10), mean (SD)			4.88 (2.53)

^a^ICU: intensive care unit.

^b^Special units include emergency rooms, operating rooms, recovery rooms, and delivery rooms.

**Table 4:** Effectiveness of intervention by variables (N=300).

Variables	Pretest score, mean (SD)	Posttest 1 score, mean (SD)	Posttest 2 score, mean (SD)	Pretest vs posttest 1			Posttest 1 vs posttest 2			Pretest vs posttest 2	
				*t (df=299)*	*P* value	*t (df=299)*		*P* value	*t (df=299)*		*P* value
Burnout^a^	62.36 (18.00)	55.22 (18.79)	52.70 (20.15)	7.012	<.001	2.811		.01	8.692		<.001
Personal^b^	20.40 (6.39)	18.15 (6.38)	17.30 (7.33)	6.134	<.001	2.597		.01	7.535		<.001
Work-related^c^	22.22 (6.39)	19.83 (6.49)	19.06 (6.68)	6.816	<.001	2.306		.02	8.286		<.001
Client-related^d^	19.72 (6.63)	17.22 (6.91)	16.32 (7.15)	6.439	<.001	2.704		.01	8.329		<.001
Job stress^e^	88.39 (12.65)	—^f^	81.85 (17.34)	—	—	—		—	6.765		<.001
Stress response^g^	42.00 (17.95)	—	35.56 (18.39)	—	—	—		—	5.864		<.001
Coping strategy^h^	70.25 (9.66)	—	70.97 (10.05)	—	—	—		—	–1.367		.17

^a^Burnout total score range is between 0 and 100.

^b^Personal burnout score range is between 0 and 31.5.

^c^Work-related burnout score range is between 0 and 37.

^d^Client-related burnout score range is between 0 and 31.5.

^e^Job stress score range is between 23 and 115.

^f^Not applicable.

^g^Stress response score range is between 0 and 88.

^h^Coping strategy score range is between 33 and 99.

For program 1, the most frequently recommended program was laughter therapy (46.3%), followed by storytelling and reflective writing (20%), ACT (18.3%), and mindfulness meditation (15.3%). For program 2, the most frequently recommended program was mindfulness meditation (41.3%), followed by laughter therapy (23.7%), storytelling and reflective writing (21.3%), and ACT (13.7%). The effect of each program in reducing burnout was as follows: laughter therapy (8.7%), storytelling and reflective writing (8.7%), ACT (12.3%), and mindfulness meditation (4.5%). Thus, among the 4 programs, ACT reduced burnout the most. Client-related burnout was decreased the most by ACT (18.2% decrease). Laughter therapy reduced personal burnout by 10.4%, which was the highest reduction. Work-related burnout was reduced the most through storytelling and reflective writing and ACT (both resulted in a reduction of 10.5%) (Table 5).

Table 6 shows the correlation with the order of participation and burnout reduction. The correlation of burnout differences between posttest 1 and pretest and the order of participation was 0.060 (*P*=.30). The correlation of burnout differences between posttest 2 and posttest 1 and the order of participation was 0.138 (*P*=.04). The correlation of job stress differences between pretest and posttest 2 and the order of participation was 0.134 (*P*=.03). The correlation of stress response differences between pretest and posttest 2 and the order of participation was 0.094 (*P*=.13). The correlation of coping strategy differences between pretest and posttest 2 and the order of participation was 0.140 (*P*=.08).

**Table 5:** Details for each program (N=300).

			Laughter therapy	Storytelling and reflective writing	ACT^a^	Mindfulness meditation
Program 1 (participants), n (%)			139 (46.3)	60 (20)	55 (18.3)	46 (15.3)
Program 2 (participants), n (%)			71 (23.7)	64 (21.3)	41 (13.7)	124 (41.3)
**Burnout reduction**						
	**Personal**					
		Score in points (%)	–6.9 (10.4)	–4.2 (8.0)	–4.9 (7.9)	–3.0 (4.9)
		*t (df)*	5.403 (209)	2.020 (123)	2.635 (95)	2.266 (169)
		*P* value	<.001	.046	.01	.03
	**Work-related**					
		Score in points (%)	–4.3 (7.2)	–5.6 (10.5)	–6.4 (10.5)	–2.2 (4.0)
		*t (df)*	4.272 (209)	3.283 (123)	3.702 (95)	1.820 (169)
		*P* value	<.001	.00	<.001	.07
	**Client-related**					
		Score in points (%)	–5.2 (8.7)	–3.8 (7.4)	–12.7 (18.2)	–2.7 (4.8)
		*t (df)*	3.718 (209)	1.870 (123)	6.897 (95)	1.977 (169)
		*P* value	<.001	.06	<.001	.05
	**Total**					
		Score in points (%)	–5.4 (8.7)	–4.6 (8.7)	–7.9 (12.3)	–2.6 (4.5)
		*t (df)*	4.833 (209)	2.546 (123)	4.886 (95)	2.241 (169)
		*P* value	<.001	.01	<.001	.03
Satisfaction, mean (SD)			4.58 (0.60)	4.69 (0.54)	4.52 (0.68)	4.61 (0.57)

^a^ACT: acceptance and commitment therapy.

**Table 6:** Correlation with the order of participation and research variables.

Stages		Order of participation
**Burnout differences between posttest 1 and pretest**		
	*R*	0.060
	*P* value	.30
**Burnout differences between posttest 2 and posttest 1**		
	*R*	–0.138
	*P* value	.04
**Job stress differences between posttest 2 and pretest**		
	*R*	–0.134
	*P* value	.03
**Stress response differences between posttest 2 and pretest**		
	*R*	–0.094
	*P* value	.13
**Coping strategy differences between posttest 2 and pretest**		
	*R*	–0.140
	*P* value	.08

### Participant Satisfaction

**Figure 4:**
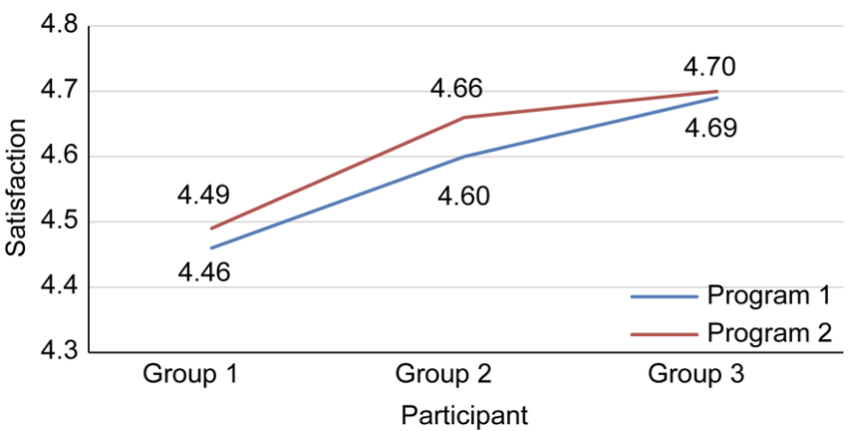
Changes in participant satisfaction. Group 1 included the first to the 100th participants, group 2 the 101st to the 200th, and group 3 the 201st to the 300th.

When programs were compared, the satisfaction score for storytelling and reflective writing (4.69 points) was the highest, followed by mindfulness meditation (4.61 points), laughter therapy (4.58 points), and ACT (4.52 points) (Table 5). The satisfaction scores increased over time from participant 1 in October 2022 to participant 300 in January 2023 by optimization (Figure 4). By grouping participants into groups of 100, we were able to calculate the average satisfaction of programs 1 and 2 to understand how satisfaction changed. For program 1, satisfaction scores for the 3 groups increased from 4.46 to 4.60 and 4.69 (*F*=3.493; *P*=.03). The post hoc test showed statistically significant differences between the first 100 and the third 100 (*P*=.03). For program 2, the satisfaction score was 4.49 points for the initial 100 participants, it was 4.66 points for the next 100 participants, and the score was 4.70 for the last 100 participants; thus, the satisfaction for program 2 also gradually increased (*F*=3.911; *P*=.02). In addition, the difference in satisfaction with the second program was statistically significant as a result of the post hoc analysis between the first 100 and the third 100 participants (*P*=.03).

## Discussion

### Principal Findings

In this study, we developed an AI-based tailored mobile app and optimized the algorithm for nurse burnout. As a result of organizing and optimizing the system with an algorithm that recommends programs that have been effective for highly similar users, burnout decreased and satisfaction scores increased as data accumulated. Furthermore, when we examined whether the algorithm was being applied correctly, we verified that the system recommends the most similar valid data to new users, proving its accuracy.

The unique aspect of this study is the introduction of AI into a program recommendation system. Thus, our system is distinct from other such programs and has unique features in terms of its development. Several studies have used AI to predict or manage burnout based on physiological indicators or to predict the degree of burnout [61-64]. Although predictions and measurements were developed, no suitable strategy for reducing burnout was presented. However, 1 study used AI in stress management programs [65]. AI algorithms were used only to guide programs and programs were conducted in group settings, thus overlooking the aspect of diversity. To the best of our knowledge, no previous study has incorporated AI to provide tailored programs to users to reduce burnout.

Our AI-based algorithm was designed to effectively reduce burnout in each user by referring to similarity. As the algorithm was optimized with an accumulation of valid data, satisfaction increased significantly. In terms of burnout reduction, it was found that the change in the burnout value from posttest 1 to posttest 2 gradually increased as the algorithm was optimized. Thus, the recommendation for the second program was more optimal for burnout reduction.

The strength of our intervention is that it is a tailored program based on user input on their burnout dimensions and demographic data. Previous studies provided general burnout programs even though dimensions of nurse burnout have long been studied. Providing tailored intervention is effective in reducing the emotional exhaustion of nurses [66]. A tailored program is an effective intervention that provides motivation to continue the program, reduces stress by increasing participant engagement, and increases participant satisfaction [67]. Therefore, traditional and conventional care is less effective while personalized care is considered the best [68]. However, interventions that provide tailored programs suited to individual characteristics are scarce in the literature. The program that we developed proved to be an effective intervention by increasing satisfaction and reducing burnout, job stress, and stress response. This program could become a pioneer and serve as a guide for the development of burnout reduction programs in the future.

Nurse Healing Space consists of visual programs in which nurse characters guide users by narrating, demonstrating the movement of body parts, showing facial expressions, and singing which might overcome the limitations of previously developed audio programs. Recent interventions that have proven to be effective for nurse burnout are commercially available audio-based programs, such as Headspace and UCLA Mindful [12,13]. However, audio-based programs through mobile apps could result in increased dropout rates due to a lack of participant concentration [69]. Moreover, a previous study demonstrated that video-based exercise programs were effective [70] and had a low dropout rate [71].

The users were highly satisfied with the app’s convenience and high-quality visuals. However, they were dissatisfied with the limitations of alarm functions and individual settings that need improvement.

CBT has long been used for burnout. In this study, among 4 CBT programs, ACT showed the greatest burnout reduction rate. This is interesting considering that the most researched program for nurse burnout is mindfulness meditation [29]. In recent years, ACT has been increasingly adopted to prevent and reduce professional staff burnout in medical and other workplace settings [50]. In addition, ACT was reported as an effective stress management intervention for medical staff, including nurses [49]. Moreover, in our study, storytelling and reflective writing and laughter therapy reduced burnout scores more effectively than mindfulness meditation. Although many studies have verified the effectiveness of a single program, further research is needed to compare the effects of different burnout programs.

Although participants experienced reduced job stress and stress responses after the intervention, no statistically significant change was reported in the coping strategy level. This could be because the coping strategies introduced in the interventions were not put into action. Conducting debriefing sessions or forming online communities to enhance behavior changes could assist users in coping.

### Limitations

We recruited 300 users to optimize the AI algorithm. The resulting sample is not truly representative of the entire population and hence the results are not generalizable [72]. Moreover, the absence of a control group restricts our capacity to determine the causal links underlying the effects of the intervention. Although the satisfaction scores for the program increased as the number of users increased, algorithm optimization is an ongoing process.

### Conclusions

In this study, we developed an AI-based tailored mobile intervention for burnout and optimized the algorithm to suggest programs to users based on user similarities. Our mobile app’s usability was acceptable, as was discovered through postchecking and satisfaction measurement , which showed that the algorithm functions normally. In addition, based on the CBI scores before and after the program, valid data were accumulated for 45.7% (274/600) of the participants. Through the development of interventions using an AI program, we showed that not only did nurse burnout decrease, job stress and stress responses also decreased. Nurses may be motivated to participate in programs to reduce burnout through interventions tailored to various forms of burnout. This program is the first customized burnout reduction program for nurses, and nurse managers can make use of AI-based systems to offer specialized programs for nurse burnout. This could reduce nurse retirement rates, improve patient safety and qualitative health care, increase employee satisfaction, reduce costs, and ultimately improve the efficiency of the health care system.

## Abbreviations

ACT: acceptance and commitment therapy
AI: artificial intelligence
CBI: Copenhagen Burnout Inventory
CBT: cognitive behavioral therapy
